# ACOMPANYA’M a novel multimodal intervention plan

**DOI:** 10.1192/j.eurpsy.2024.1243

**Published:** 2024-08-27

**Authors:** I. Insa Pineda, I. Rueda Bárcena, C. Lamborena Ramos, F. de Pedro Melgarejo, A. Planas Bas, T. Prieto Toribio, E. Lujan Lujan, M. Ribas Siñol

**Affiliations:** ^1^Child and Adolescent mental Health Area; ^2^Child and Adolescent Mental Health Research Group, Hospital Sant Joan de Déu, Esplugues de Llobregat; ^3^Mental Health, Parc Sanitari Sant Joan de Déu, Sant Boi de Llobregat, Spain

## Abstract

**Introduction:**

The Residential Educational Therapeutic Unit Accompany from Hospital Sant Joan de Déu Barcelona, is a device integrated into the public health network, intended for the comprehensive care of **children and adolescents under 18 years of age who suffer from an illness complex mental disorder**, at serious risk of becoming chronic and generating significant disabilities at a functional, cognitive and emotional level. It was a result from a joint venture between the Department of Social Rights and the Department of Health. The device was created to respond to the increase in behavioral problems and mental health disorders of children underguardianship.

**Objectives:**

**General Objective**

To improve the quality of life in the physical, mental and social spheres of vulnerable children and adolescents with serious complex mental pathology through a biopsychosocial and community care model that integrates health, social, family and educational care and which is aimed at the recovery of the person’s life project.

**Specific Objectives**

To offer intensive intervention, personalized and in a co-responsible manner, that is to say, that integrates the therapeutic, education, social services and child protection teams.

Promote the community and social reintegration avoiding stigmatization and social exclusion.

Improve the intra-family relationship and the burden perceived by caregivers.

Decrease the number of renunciations of parental authority of a minor.

**Methods:**

The unit has a capacity for **28 beds:** 23 places for children/adolescents underguardianship of the administration and 5 places for cases that are at risk of family claudication due to their therapeutic and educational needs.

There are 5 coexisting therapeutic units. The apartments are referred as ‘homes’ and their organization is designed to encourage the active participation of residents with the professionals who attend them.

**The Unit has a multidisciplinary team made up of the following professionals:** Psychyatrists, Nurses, clinical psychologists, Social Workers, Educational worker, nursing assistants, administrative.

**Results:**

- **110 children and adolescents have been taken care**, with an **average cumulative stay of 13 months**. In all cases in which the family had the guardianship of the patient, family claudication has been avoided There is a 36% discharge of those patients under guardian that have returned to their original family home 100% of the cases have been linked to an educational center adapted to their needs or to a training project

**Image:**

**Figure:**
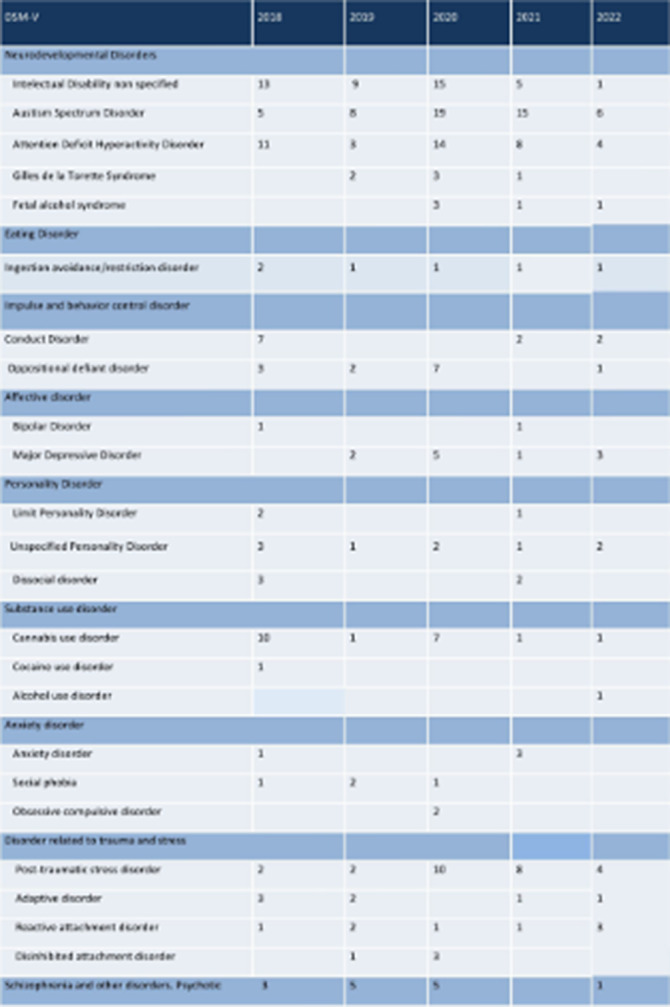

**Image 2:**

**Figure:**
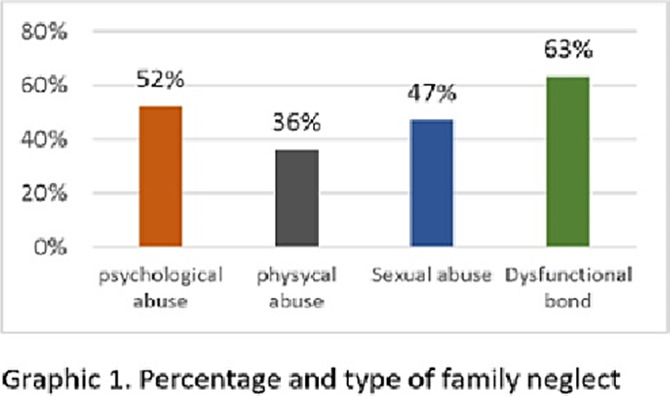

**Conclusions:**

Overall, the care model implemented by the population served in the Acompanya’m unit is positively evaluated. Since it provides an intensive and personalized care, treatment and intervention for children suffering from a serious mental disorder of high complexity. A comprehensive, personalized, interdisciplinary approach is offered, coordinated and co-responsible with educational, protection and social services.

**Disclosure of Interest:**

None Declared

